# Overexpression of FGF2 delays the progression of osteonecrosis of the femoral head activating the PI3K/Akt signaling pathway

**DOI:** 10.1186/s13018-021-02715-9

**Published:** 2021-10-18

**Authors:** Pei Lu, Yi-min Shen, Ting Hua, Ting Pan, Gang Chen, Teng Dai, Ke-qin Shi

**Affiliations:** Department of Orthopaedics, The Affiliated Wuxi No.2 People’s Hospital of Nanjing Medical University, No. 68 Zhongshan Road, Wuxi City, 214000 Jiangsu Province China

**Keywords:** FGF-2, Bioinformatics analysis, Apoptosis, PI3K, Akt

## Abstract

**Background:**

The purpose of the current study was to explore the role and underlying mechanism of FGF-2 in dexamethasone (DEX)-induced apoptosis in MC3T3-E1 cells.

**Methods:**

GSE21727 was downloaded from the Gene Expression Omnibus (GEO) database to identify the differentially expressed genes (DEGs) by the limma/R package. MC3T3-E1 cells were exposed to DEX at different concentrations (0, 10^−8^, 10^−7^, 10^−6^, 10^−5^ and 10^−4^ mol/L), and cell viability, flow cytometry and TUNEL assay were used to detect cell proliferation and apoptosis. An FGF-2-pcDNA3 plasmid (oe-FGF-2) was used to overexpress FGF-2, and western blotting was conducted to detect protein expression.

**Results:**

We found that FGF-2 was downregulated in the DEX-treated group. Kyoto Encyclopedia of Genes and Genomes (KEGG) pathway analyses indicated that DEGs were associated with PI3K/Akt signaling pathway. DEX downregulated FGF-2 gene and protein expression, inhibited viability and induced MC3T3-E1 cell apoptosis. Overexpression of FGF-2 reversed DEX-induced apoptosis in MC3T3-E1 cells. FGF-2-mediated anti-apoptosis was impaired by inactivating the PI3K/AKT pathway with LY294002. Moreover, overexpression of FGF2 delayed the progression of DEX-induced osteonecrosis of the femoral head (ONFH) animal model by regulation PI3K/Akt signaling pathway.

**Conclusion:**

In conclusion, FGF-2 is effective at inhibiting DEX-induced MC3T3-E1 cell apoptosis through regulating PI3K/Akt signaling pathway.

**Supplementary Information:**

The online version contains supplementary material available at 10.1186/s13018-021-02715-9.

## Introduction

Glucocorticoids (GCs), first-line anti-inflammatory agents, are widely used in inflammation and autoimmune diseases due to their strong anti-inflammatory effects [1–3]. After megadose or long-term application of GCs, patients are at risk of osteoporosis or even osteonecrosis of the femoral head (ONFH) [4–7]. Kubo et al. [8] found that approximately 51% of cases of osteonecrosis of the femoral head were associated with GC intake. However, the exact pathogenesis of GCs mediating impaired bone formation remains elusive [9]. Many scholars have indicated that the increase in osteoblast apoptosis is one of the important mechanisms of GC-induced bone loss [10, 11].

Fibroblast growth factors (FGFs) are secreted glycoproteins and possess many biological functions, such as regulating cell viability and apoptosis [12]. To date, a total of 22 ligands of FGF families have been discovered, and their function is mainly through binding to FGF receptors (FGFRs) [13]. Byun et al. [14] observed that TAZ mediated FGF2 signaling and promoted osteogenic differentiation of C3H10T1/2 cells. Montero et al. [15] found that by disruption of the FGF2 gene in mice, bone mass and bone formation were decreased. Moreover, FGF2 attenuates neuronal apoptosis after subarachnoid hemorrhage [16].

Gene expression microarrays have been widely used to study gene expression in many diseases [17]. Bioinformatics analysis was performed to identify the key regulatory mediator of MC3T3-E1 cell apoptosis and an in vitro study was conducted to test this hypothesis. Our findings provide novel insights into the mechanism underlying DEX-induced apoptosis of MC3T3-E1 cells, and FGF-2 may be a new target for ONFH.

## Materials and methods

### Microarray analysis

The mRNA expression profile GSE21727 was retrieved from the GEO database (https://www.ncbi.nlm.nih.gov/geo/). In this microarray, 3 samples of primary human osteoblasts were treated with dexamethasone (DEX, 10^−4^ mol/L) for 24 h and 6 samples without treatment as controls. Differentially expressed genes (DEX-treated vs Con) were further analyzed by the R package ‘Limma’ from the Bioconductor project [18]. |logFC|> 1 and *P* value < 0.05 was set as the cutoff point.

### Function enrichment analysis

Gene Ontology (GO) analysis, which includes biological processes (BP), cellular components (CC) and molecular function (MF), and Kyoto Encyclopedia of Gene and Genomes (KEGG) pathway enrichment analysis, was carried out using the clusterProfiler (version 3.10.1) package [19]. DOSE packages in R software were used to visualize the GO and KEGG results [20]. Differences were considered statistically significant when *P* < 0.05. We used the Search Tool for Interactions of Chemicals (STITCH, http://stitch.embl.de/) online database to predict and construct the biological network of DEX [21]. An interaction score > 0.4 was considered statistically significant.

### Cell culture and plasmid transfection

The mouse preosteoblast cell line MC3T3-E1 was obtained from American Type Culture Collection (ATCC, CRL-2593) and maintained in DMEM (Gibco, Life Technologies, Carlsbad, CA, USA) supplemented with 10% FBS (Gibco, Life Technologies, Carlsbad, CA, USA), 10 mM HEPES (Sigma-Aldrich, Poole, UK), and 0.1% penicillin–streptomycin (Sigma-Aldrich, Poole, UK). The FGF-2-pcDNA3 plasmid (oe-FGF-2) was synthesized by GeneChem, Inc. Then, MC3T3-E1 cell suspensions (150 μl, containing 1 × 10^4^ cells) were plated onto the cover glass of a confocal petri dish (NEST, Hong Kong, China) for transient transfection. DMEM (1 mL) containing 10% FBS was added to the dish, and the cells were cultured for 24 h prior to transfection. Transfection was performed using Lipofectamine 3000 (Thermo Fisher Scientific) and Opti-MEM reduced-serum media (Life Technologies, Waltham, Massachusetts, USA) according to the manufacturer’s instructions. To explore the mechanisms of FGF-2-mediated MC3T3-E1 cell apoptosis, the PI3K inhibitor LY294002 was used to pretreat cells (20 μM, MCE, Shanghai, China) for 2 h followed by stimulation with FGF-2-pcDNA3 for 12 h. The choice of inhibitor concentrations and time course was based on a previous study [22].

### Cell viability assay

MC3T3-E1 cells (5 × 10^3^ per well, 200 μl) were seeded in 96-well culture plates. Then, cells were exposed to different doses of DEX (0, 10^−8^, 10^−7^, 10^−6^, 10^−5^ and 10^−4^ mol/L, Sigma-Aldrich, CAS, 50-02-2) to identify the optimal dose to induce apoptosis of MC3T3-E1 cells. After 24 h, cell viability was measured using Cell Counting Kit-8 Assay (CCK-8, Solarbio, Beijing, China) following the manufacturer’s protocol.

### Apoptosis flow cytometry assay

MC3T3-E1 cell apoptosis was assessed using Annexin V-fluorescein isothiocyanate (FITC)/propidium iodide (PI) double staining following the manufacturer’s instructions (Haime Jiangsu, China). After exposure to DEX for 24 h, MC3T3-E1 cells were collected in a centrifuge tube and stained with 5 μl Annexin V-FITC (10 μg/mL) and 5 μl PI (5 μg/mL) for 30 min. After washing with phosphate-buffered saline (PBS) three times, the cells were analyzed with a FACSCalibur flow cytometer (BD Biosciences). At least 50,000 events were detected and analyzed for apoptotic cells. Apoptosis rate was calculated as ratio of apoptotic cells in Q3 + Q2 to total cells.

### Terminal deoxynucleotidyl transferase dUTP nick end labeling (TUNEL) assay

For TUNEL staining, MC3T3-E1 cells were rinsed with PBS for 5 min and fixed with 4% paraformaldehyde for 1 h. Then, MC3T3-E1 cells were permeabilized with 0.1% Triton X-100 for 10 min. An In Situ Cell Death Detection Kit (Roche) was used to incubate the cells for 1 h at 37 °C in the dark. Finally, MC3T3-E1 cells were incubated with DAPI (Solarbio Biotechnology, Beijing, China) at room temperature in the dark for 5 min. High-resolution images were captured using an Olympus CX41 microscope (Center Valley, PA, USA). The percentage of TUNEL-positive MC3T3-E1 cells was calculated by counting TUNEL-positive cells/total number of cells (%) in 5 random high-power fields.

### Reverse transcription PCR assay

Total RNA of MC3T3-E1 cells was extracted using the TRIzol method as previously described [23]. cDNAs were synthetized by reverse transcription using oligo(dT) with RNA samples. cDNAs were amplified by a SYBR Green PCR kit. The relative level of FGF-2 was normalized to the GAPDH level. The expression of FGF-2 and GAPDH was detected using qPCR with SYBR Green Mix Kits (Applied Biosystems). All results were quantitated using the 2^−ΔΔCt^ relative quantification method. The primer pair sequences were as follows: GAPDH forward, 5′-AAG GCC ATC ACC ATC TTC CA-3′, GAPDH reverse, 5′-GGA TGC GTT GCT GAC AATCT-3′; FGF-2 forward, 5′-CGAGTGAGAGGCAACTTGG-3′, FGF-2 reverse 5′-CGGTTACAGAACCAC ACACG-3′.

### Western blot analysis

Total protein was extracted from MC3T3-E1 cells for western blot analysis. Briefly, cells were immersed in RIPA buffer and then separated by sodium dodecyl sulfate–polyacrylamide gel electrophoresis (SDS-PAGE). The separated proteins were transferred onto polyvinylidene difluoride (PVDF) membranes. The membranes were blocked with 5% nonfat milk dissolved in Tris-buffered saline with 0.05% Tween-20 (TBS-T) for 1 h at room temperature. The membranes were then incubated overnight at 4 °C with primary antibodies against cleaved Caspase 3 (Cell Signaling; Beverly, MA, #9664, 1:1000), Caspase 3 (Cell Signaling; Beverly, MA, #9662, 1:1000), phospho-Akt (Ser473, Cell Signaling, Beverly, MA, #4060, 1:2000), phospho-PI3K (Tyr458, Cell Signaling, Beverly, MA, #9655,1:1000), Akt (Cell Signaling, Beverly, MA, #4685, 1:2000), PI3K (Cell Signaling, Beverly, MA, #4249, 1:1000), FGF-2 (Abcam, Cambridge, UK, ab8880, 1:5000), Bax (Abcam, Cambridge, UK, ab32503, 1:2000), Bcl-2 (Abcam, Cambridge, UK, ab182858, 1:5000), and GAPDH (Proteintech, Wuhan, China, 6004-1-1g 1:6000). Finally, the membranes were incubated for 1 h with horseradish peroxidase (HRP)-conjugated secondary antibodies (1:500) and visualized using an enhanced chemiluminescence system, according to the manufacturer’s instructions.

### Animal studies

All animal experiments were performed following the guidelines for experimentation with laboratory animals set in The Affiliated Wuxi No.2 People’s Hospital of Nanjing Medical University. ONFH animal model were established based on method published previously [24]. Fifteen male Sprague–Dawley rats (weighing 250–300 g) were randomly divided into two groups: (1) Normal groups (n = 5), (2) DEX (Dex 5 mg/kg.d, n = 5)-treated group, (3) Dex + FGF-2-pcDNA3 group (Dex 5 mg/kg.d, n = 5). In brief, FGF-2-pcDNA3 was injected into medullary cavity of bilateral distal femur once a week. All rats were housed under specific pathogen-free conditions with free access to food pellets and tap water for 1 month.

### Hematoxylin and eosin (HE) staining and immunohistochemistry (IHC)

Femoral heads in each group were harvested and fixed with 4% paraformaldehyde for 1 day and processed for paraffin embedding. Following embedding in paraffin, samples were cut into 5-μm sections. Subsequently, the sections were dewaxed and hydrated, followed by HE staining according to the instruction manual. Following deparaffinization, epitope retrieval was performed in a citrate buffer (pH 6.0) heated in a microwave oven for 10 min. The sections were incubated with 20% BSA blocking solution (Solarbio, Beijing, China, 37 °C, 30 min) for blocking non-specific staining. The slides were then incubated with p-Akt primary antibody (Cell Signaling, Cat#4060S, RRID: AB_2315049) at 4 °C overnight, followed by incubation with biotin-conjugated secondary antibody (1:1000; sc-2004; Santa Cruz Biotechnology, Inc.). Photographs were taken with an Olympus Optical AX70 microscope (Olympus).

### Statistical analysis

All statistical analyses were performed using SPSS1 version 7.0 software. The data are expressed as the mean ± standard deviation (SD, n = 3). Student’s t test or one-way analysis of variance was conducted to compare differences between groups. *P < 0.05 was considered to be statistically significant.

## Results

### Identification of differentially expressed genes

**Fig. 1:**
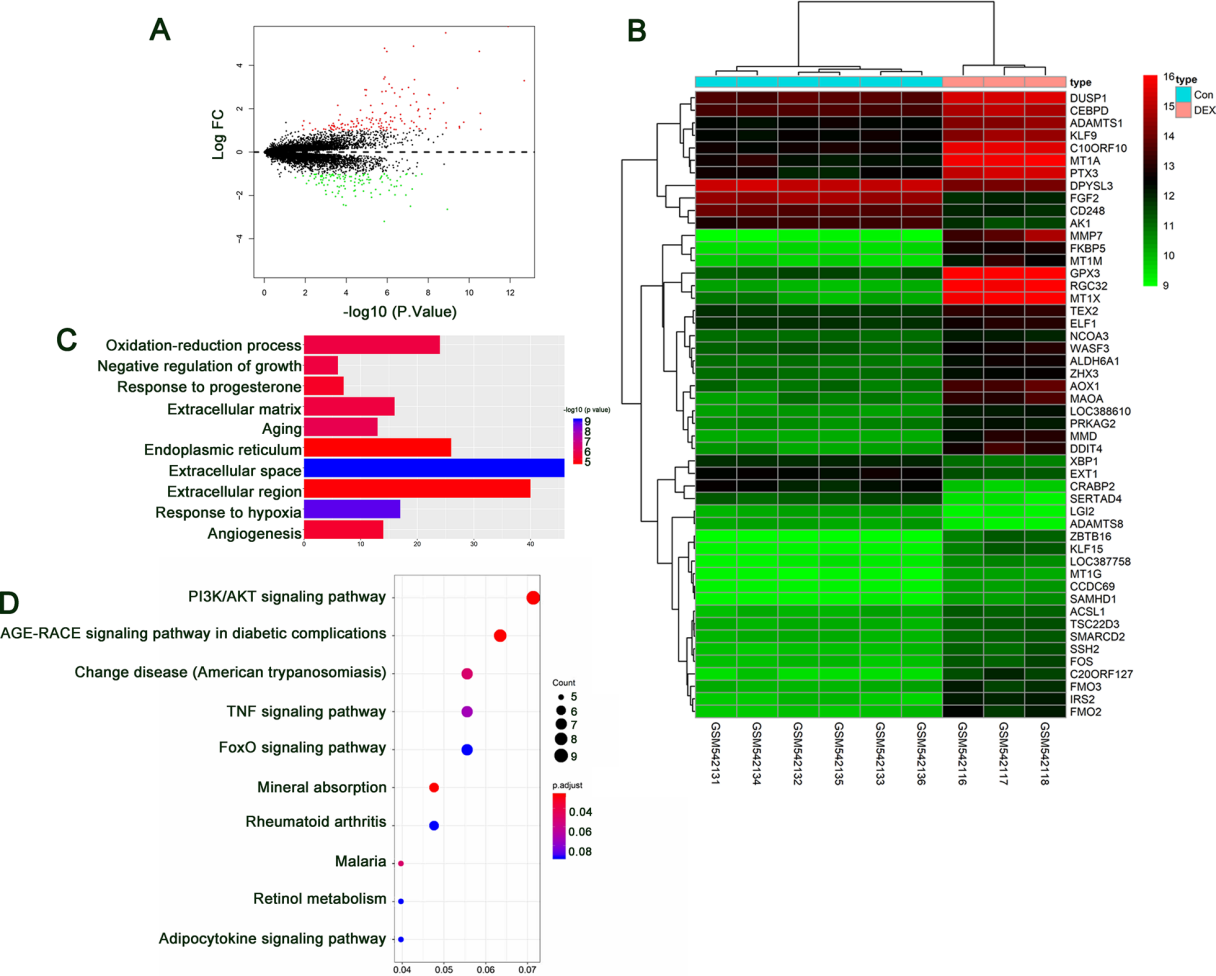
Identification of DEGs in human osteoblasts exposed to DEX. **a** Volcano plot analysis of 258 DEGs. The red dots represent the upregulated DEGs, green dots represent downregulated DEGs, and black dots represent genes with no significant change in expression. **b** Heatmap graphs of the DEGs in the experimental groups: DEX versus control. Arbitrary signal intensity acquired from the microarray analysis is represented by the colors (green = lower expression; red = higher expression). Log2 signal intensity values for any single gene were resized to row *Z*-score scales. **c** Top 10 significantly enriched GO terms of DEGs. The x-axis represents the − log10 (*P* value) associated with GO terms, and the y-axis represents the significantly enriched GO terms. **d** A bubble chart showing the identified KEGG pathways. The abscissa represents the GeneRatio, and the ordinate represents the KEGG pathway terms. *P* values indicate larger values from blue through to red, and a larger node size represents a higher number of enriched genes

The selection criteria were set as |logFC|> 1 and *P* value < 0.05 for the selection of DEGs. A volcano plot of the identified DEGs is shown in Fig. 1a, and a heatmap of the DEGs is shown in Fig. 1b. A total of 258 DEGs were identified, among which 117 DEGs were downregulated, while 141 DEGs were upregulated. We found that FGF-2 was downregulated in the DEX-treated group. To further understand the function and mechanism of the identified DEGs, GO enrichment analyses were performed using the ClusterProfiler package. Figure 1c presents the top ten significantly enriched GO terms. The DEGs were mainly associated with oxidation–reduction process and negative regulation of growth. The top 10 DEG-enriched pathways are represented by a bubble chart and shown in Fig. 1d.

### FGF-2 is downregulated by DEX and its related biological functions

**Fig. 2:**
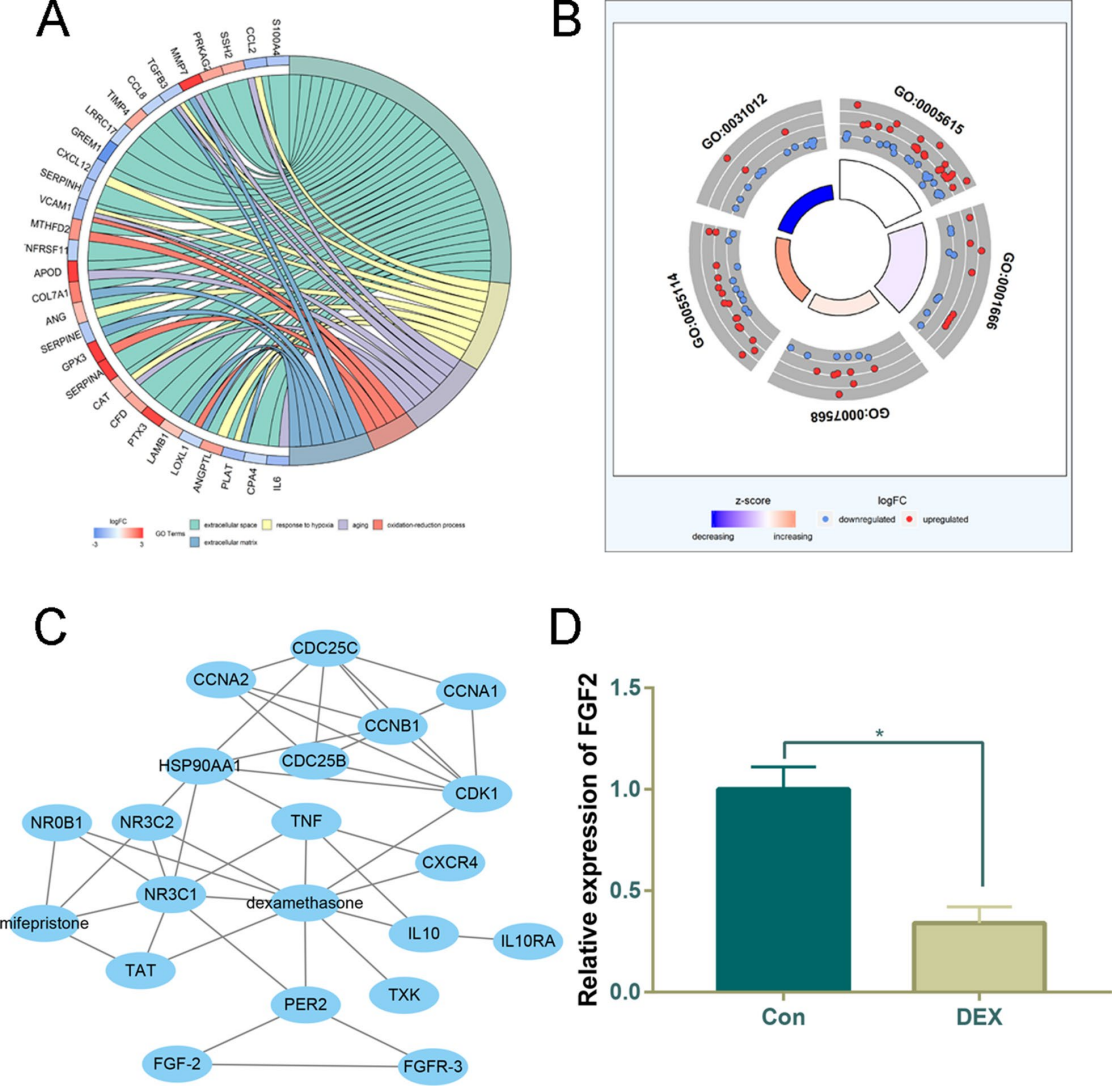
**a** Chord diagram that presents the DEGs linked via ribbons to their assigned GO terms. Color coding indicates logFC of upregulated genes (red) or downregulated genes (blue). **b** GO circle plot; the inner color indicates the significance of the term (*z*-score), where pink represents increasing and purple represents decreasing. The outer ring displays scatterplots of the expression levels (logFC) for the genes in each GO term. **c** DEX-protein interaction network based on STITCH database. **d** FGF-2 expression in primary MC3T3-E1 cells from control or DEX after 24 h treatment. *P < 0.05, *DEX* dexamethasone

FGF-2 was significantly downregulated after DEX treatment. FGF-2 and its adjacent gene-related GOs are presented in a chord diagram (Fig. 2a) and a circle plot (Fig. 2b). From the STITCH database, a network involving DEX and its interacting proteins was constructed, and we observed that FGF-2 was not a direct target gene of DEX but was closely related to PER2, a direct target gene of DEX (Fig. 2c). To validate our results, we performed an in vitro experiment, and the qRT-PCR results further confirmed that DEX could decrease FGF-2 mRNA expression (Fig. 2d).

### DEX promotes apoptosis of MC3T3-E1 cells

**Fig. 3:**
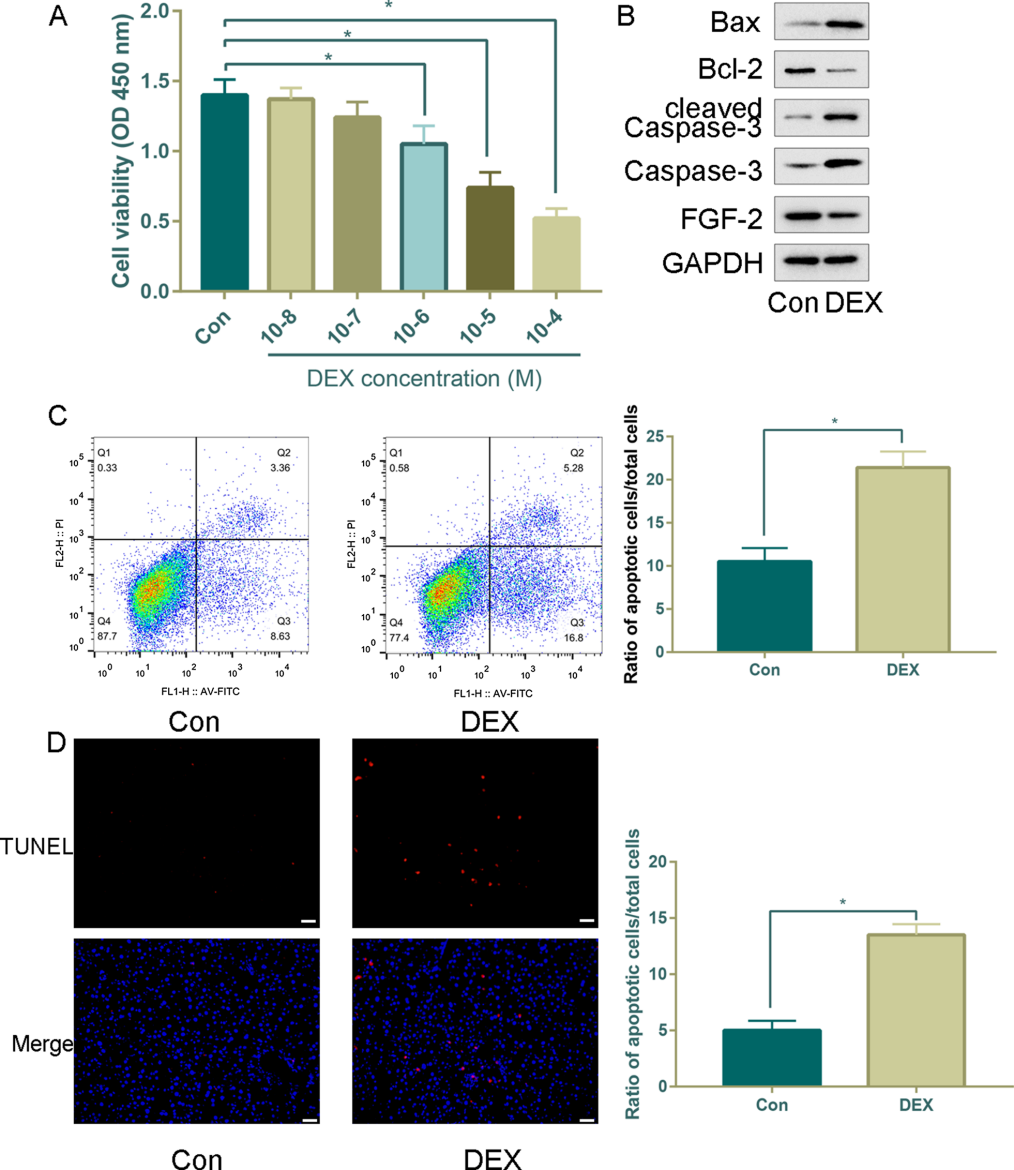
The apoptosis assay revealed that DEX promoted cell apoptosis in MC3T3-E1 cells. **a** Effects of DEX on MC3T3-E1 cell proliferation, as demonstrated by CCK-8 assay. **b** Protein levels of Bax, Bcl-2, Cleaved caspase-3, Caspase-3 and FGF-2 were detected by western blotting after DEX treatment. **c** Flow cytometry after Annexin V/PI staining and quantitative analysis of the apoptosis ratio after DEX treatment. **d** Apoptosis by TUNEL staining after DEX treatment (× 100 magnification); *P < 0.05, *DEX* dexamethasone

DEX significantly decreased the viability of MC3T3-E1 cells in a dose-dependent manner, with the maximal concentration that induced MC3T3-E1 apoptosis being 10^−4^ mol DEX (Fig. 3a). Therefore, 10^−4^ M DEX was used for subsequent studies based on our results and GSE21727. Consistent with the change in FGF-2 mRNA expression, the FGF-2 protein level was much lower in the DEX group than in the control group (Fig. 3b). DEX induced an increase in Bax, Cleaved caspase-3 and Caspase3 (proapoptotic protein) and a decrease in Bcl-2 (antiapoptotic protein) (Fig. 3b).

To further explore the impact of DEX on MC3T3-E1 cell apoptosis, we used Annexin V/PI double staining and TUNEL assays to examine apoptosis. The apoptosis ratio was increased by 10.8% (Fig. 3c), indicating the promoting role of DEX in MC3T3-E1 cell apoptosis. We further examined and quantitated this observation using TUNEL staining (Fig. 3d). Through TUNEL assays, we observed that DEX increases the number of apoptotic cells.

### Overexpression of FGF-2 rescued DEX-induced apoptosis in MC3T3-E1 cells

**Fig. 4:**
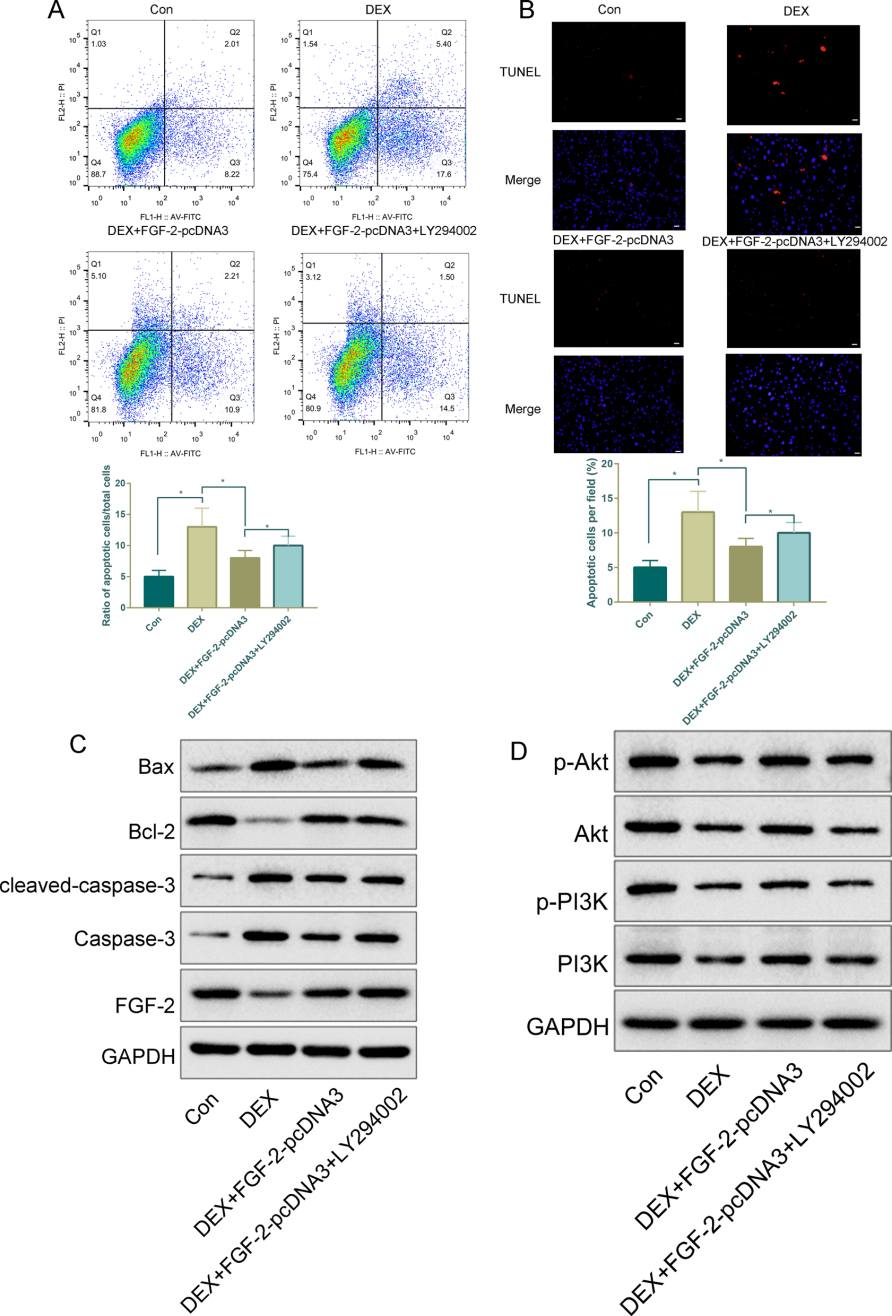
The effects of FGF-2 overexpression on DEX-induced apoptosis of MC3T3-E1 cells. MC3T3-E1 cells were pretreated with an FGF-2 expression plasmid and/or PI3K inhibitor (LY294002) 12 h before DEX treatment. Flow cytometry after Annexin V/PI staining (**a**) and TUNEL (× 100 magnification, **b**) analysis of apoptosis after DEX (10^−6^ M, 24 h), DEX + FGF-2 pcDNA3, and DEX + FGF-2 pcDNA3 + LY294002 treatments. **c** Western blotting was performed to analyze the expression levels of Bax, Bcl-2, cleaved Caspase-3, Caspase-3, FGF (**c**), PI3K, p-PI3K, AKT and p-AKT (**d**) in MC3T3-E1 cells after DEX (10^−6^ M, 24 h), DEX + FGF-2 pcDNA3, and DEX + FGF-2 pcDNA3 + LY294002 treatments. *P < 0.05, *DEX* dexamethasone

To further verify that FGF-2 is involved in DEX-induced apoptosis, FGF-2-pcDNA3 plasmid transfection for FGF-2 overexpression was used in further studies. The results showed that the expression of FGF-2 was upregulated obviously following transfection with pcDNA-FGF-2 compared with transfection with empty vector, which indicated that FGF-2 overexpression was performed successfully (Additional file 1: Fig. S1). Apoptosis was analyzed by flow cytometry using Annexin V/PI staining. As illustrated in Fig. 4a, the number of apoptotic cells in the DEX group was markedly increased after incubation for 24 h, which was partly reversed by FGF-2-pcDNA3 (*P* < 0.05). To test whether this inhibition could be reversed by the inhibition of the PI3K/Akt signaling pathway, we treated MC3T3-E1 cells with the specific inhibitor LY294002 and found that the inhibition of apoptotic MC3T3-E1 cells was increased to a certain extent. The TUNEL staining analysis provided similar results to those of Annexin V/PI staining (Fig. 4b).

Therefore, we constructed an FGF-2 overexpression plasmid and confirmed the overexpression efficiency by western blot (Fig. 4c). The protein level of FGF-2 was significantly increased in FGF-2-overexpressing MC3T3-E1 cells.

The upregulation of apoptosis-related proteins (Bax, Cleaved caspase-3 and Caspase3) induced by DEX was reversed by FGF-2-pcDNA3, as shown by western blot analysis, while a marked increase in Bcl-2, an apoptosis-related protein, was observed in FGF-2-pcDNA3 compared with DEX using western blot analysis (Fig. 4c).

It is evident from Fig. 4d that PI3K and Akt activation was significantly reduced after incubation with DEX, and FGF-2 overexpression partially restored both PI3K and Akt phosphorylation. Pretreatment with LY294002 of DEX and FGF-2-pcDNA3-treated MC3T3-E1 cells significantly decreased the expression of p-PI3K and p-Akt compared with administration of DEX and FGF-2-pcDNA3 alone. These data suggested that FGF-2 restored the activation level of the PI3K/Akt signaling cascades blocked by LY294002 in MC3T3-E1 cells.

### Overexpression of FGF-2 delayed progression of OFNH in animal model

**Fig. 5:**
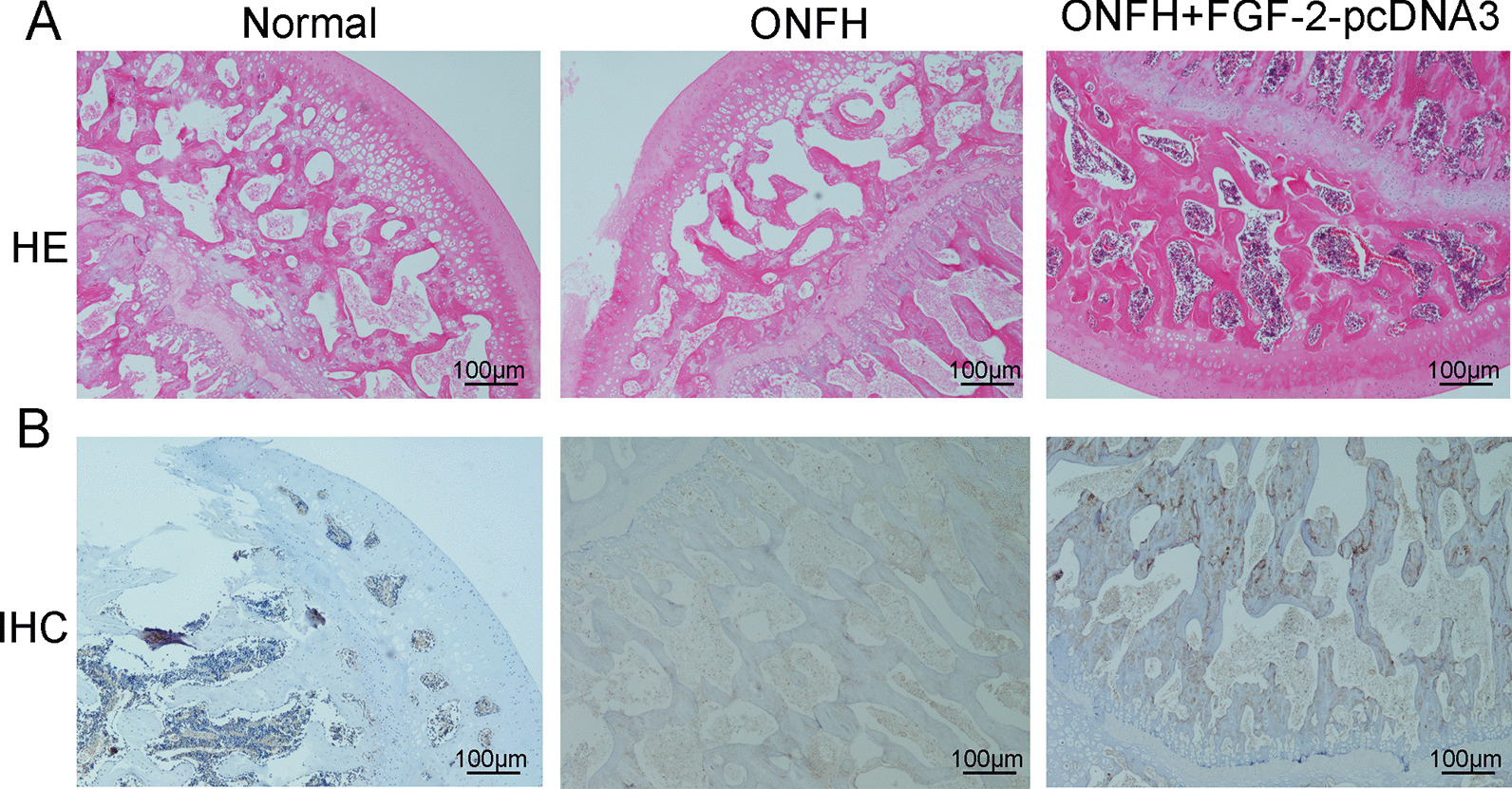
The effects of FGF-2 overexpression on delaying progression in ONFH animal model. **a** HE staining was measured histological changes in the trabecular bone microstructure after administration with FGF-2 overexpression plasmid. **b** Immunohistochemistry results of the p-Akt expression in femoral head after administration with FGF-2 overexpression plasmid (× 100 magnification)

In the ONFH group, trabecular bone destruction and fat vacuoles were significantly increased than normal group, suggesting that the ONFH animal model was successfully established. Administration with overexpression of FGF-2 could prevented the trabecular bone destruction and formulation of fat vacuoles (Fig. 5a). What is more, treatment with overexpression of FGF-2 plasmid was able to reverse downregulation p-Akt protein expression in DEX-induced animal mode (Fig. 5b).

## Discussion

Osteoblasts are bone-building cells, and the fine regulation of osteogenic differentiation is critical to the process of bone formation, modeling, and remodeling. Better understanding of the signaling pathways involved in osteogenic differentiation may result in the discovery of novel potential targets of osteoporosis. The most common risk factors for osteoporosis include age, menopause-associated hormone changes in women, changes in physical activity, medications, and certain diseases. It is widely accepted that age-associated growth hormone, estrogens, and other hormones play a key role in the maintenance of bone homeostasis and the development of osteoporosis. DEX is a common GC with strong anti-inflammatory activity. However, large doses and long treatment courses of DEX administration may lead to osteoporosis or even ONFH. Studies have found that DEX promotes apoptosis in a variety of cells, including MLO-Y4 [25], MC3T3-E1 [26], bone marrow-derived mesenchymal stem cells (BM-MSCs) [27], and primary osteoblasts [28]. However, the underlying mechanisms have not yet been comprehensively investigated.

Previous related research studies have shown similar biological processes of osteoblasts exposed to DEX [29, 30]. Period2 (PER2) is a circadian gene and plays an important role in regulating cells apoptosis [31]. Honma et al. [32] found that PER2 is a directly target gene of DEX. Abe et al. [33] revealed that PER2 is required for the maturation of bone tissue. In this study, we suggested that PER2 directly regulating FGF2 through STITCH network. FGF-2 plays a central role in osteoblast differentiation and osteoblast survival [34]. FGF-2 has a positive role in preventing apoptosis in multiple cells [35, 36]. Analysis in the STITCH database indicated that DEX affects FGF-2 expression mainly by regulating other neighboring genes (FGFR-3 and PER2). A mechanistic study revealed that overexpression of FGF-2 activated the PI3K signaling pathway.

DEX reduced the number of MC3T3-E1 cells in a concentration-dependent manner. We used 10^−4^ M DEX to treat MC3T3-E1 cells based on our experiment and GSE21727 data [37]. The MC3T3-E1 apoptotic rate increased in the DEX group, which was determined by TUNEL staining and FITC/PI double staining by flow cytometry. After DEX treatment, the expression of apoptotic markers (Cleaved caspase-3, Caspase-3 and Bax) was increased, and the expression of an inhibitor of apoptotic proteins (Bcl-2) was decreased.

The apoptotic effect of DEX has been demonstrated in previous studies [38, 39]. FGF-2-pcDNA was used to increase the expression of FGF-2 and elucidate the underlying mechanism of FGF-2. FGF-2 played an important role in DEX-induced apoptosis, and the main result showed that overexpression of FGF-2 could rescue DEX-induced apoptosis in MC3T3-E1 cells. The apoptotic effects of FGF-2 overexpression were attenuated by blocking with a PI3K inhibitor (LY294002). The PI3K/Akt signaling pathway plays a significant role in maintaining cell viability and enhances resistance to cell apoptosis [40, 41]. Activation of the PI3K/Akt signaling pathway significantly inhibits apoptosis-related proteins [42–44].

PI3K-Akt signaling pathways showed the most significant upregulation after FGF-2 overexpression. Thus, we propose that FGF-2 effectively inhibits DEX-induced MC3T3-E1 cell apoptosis through activation of the PI3K/Akt signaling pathway. In the future, gene knockdown of FGF-2 rats should be constructed to further investigate the effects of FGF-2 for ONFH. A limitation should be noted in this study. FGF-2 silencing or PI3K/Akt direct stimulation was not perform in vivo and in vitro.

## Conclusion

In conclusion, we first conducted a bioinformatics analysis of the DEGs of control and DEX-treated human osteoblasts. We determined that FGF-2 overexpression could reverse DEX-induced apoptosis in MC3T3-E1 cells through the PI3K/Akt signaling pathway. These outcomes indicate the value of FGF-2 as a potential therapeutic target for ONFH.

## Supplementary Information

**Additional file 1. Fig. S1**: Overexpression plasmids of FGF-2 is successfully constructed. Western blot assay was performed to assess the FGF-2 expression in control and FGF-2-pcDNA3 groups.

## Data Availability

We state that the data will not be shared since all the raw data are present in the figures included in the article.

## Abbreviations

DEX: Dexamethasone
GEO: Gene Expression Omnibus
DEGs: Differentially expressed genes
oe-FGF-2: FGF-2-pcDNA3 plasmid
KEGG: Kyoto Encyclopedia of Genes and Genomes
ONFH: Osteonecrosis of the femoral head
GCs: Glucocorticoids
FGFs: Fibroblast growth factors
FGFRs: FGF receptors
GO: Gene ontology
BP: Biological processes
CC: Cellular components
MF: Molecular function
STITCH: Search Tool for Interactions of Chemicals
FITC: Annexin V-fluorescein isothiocyanate
PI: Propidium iodide
TUNEL: Terminal deoxynucleotidyl transferase dUTP nick end labeling
SDS-PAGE: Sodium dodecyl sulfate–polyacrylamide gel electrophoresis
PVDF: Polyvinylidene difluoride
TBS-T: Tris-buffered saline with 0.05% Tween-20
HE: Hematoxylin and eosin
IHC: Immunohistochemistry
SD: Standard deviation
BM-MSCs: Bone marrow-derived mesenchymal stem cells
PER2: Period2
