# Supplementary figures and images for: Overexpression of FGF2 delays the progression of osteonecrosis of the femoral head activating the PI3K/Akt signaling pathway
Source: J Orthop Surg Res. 2021 Oct 18;16:613. doi: 10.1186/s13018-021-02715-9 (PMC8522004; doi:10.1186/s13018-021-02715-9)

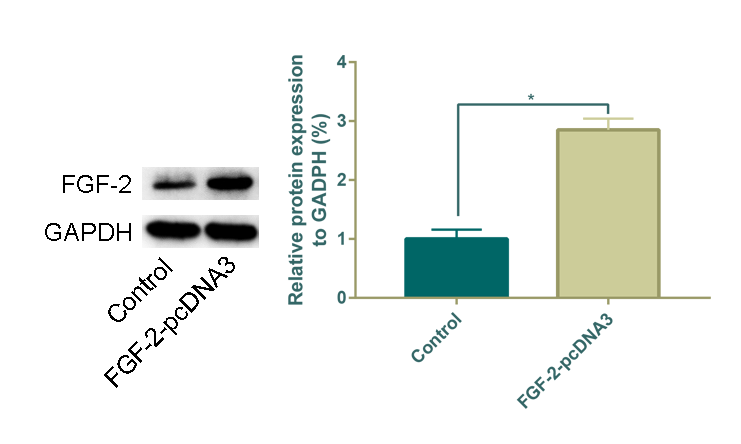

Supplement: Supplementary file 1 — Additional file 1. Fig. S1: Overexpression plasmids of FGF-2 is successfully constructed. Western blot assay was performed to assess the FGF-2 expression in control and FGF-2-pcDNA3 groups. [file 13018_2021_2715_MOESM1_ESM.tif]
